# Molecular Heterogeneity in Glioblastoma: Potential Clinical Implications

**DOI:** 10.3389/fonc.2015.00055

**Published:** 2015-03-03

**Authors:** Nicole Renee Parker, Peter Khong, Jonathon Fergus Parkinson, Viive Maarika Howell, Helen Ruth Wheeler

**Affiliations:** ^1^Bill Walsh Translational Cancer Research Laboratory, Kolling Institute of Medical Research, Royal North Shore Hospital, University of Sydney, St Leonards, NSW, Australia; ^2^Department of Neurosurgery, Royal North Shore Hospital, St Leonards, NSW, Australia; ^3^Department of Medical Oncology, Royal North Shore Hospital, St Leonards, NSW, Australia

**Keywords:** glioma, glioblastoma, heterogeneity, molecular, intra-tumoral heterogeneity, biomarkers, transcriptional subtype

## Abstract

Glioblastomas, (grade 4 astrocytomas), are aggressive primary brain tumors characterized by histopathological heterogeneity. High-resolution sequencing technologies have shown that these tumors also feature significant inter-tumoral molecular heterogeneity. Molecular subtyping of these tumors has revealed several predictive and prognostic biomarkers. However, intra-tumoral heterogeneity may undermine the use of single biopsy analysis for determining tumor genotype and has implications for potential targeted therapies. The clinical relevance and theories of tumoral molecular heterogeneity in glioblastoma are discussed.

## Introduction

Malignant gliomas are the most common intrinsic primary brain tumors in adults. Despite advances in neurosurgery, chemotherapy, and radiation, the median survival for the most aggressive tumors, glioblastoma remains less than 2 years. Although glioblastomas share common histological features, at a molecular level these tumors are highly variable from patient to patient and within the same tumor can display significant regional heterogeneity. Molecular analysis of a single biopsy specimen for diagnosis and determination of therapeutic options has profound clinical implications for targeted therapeutic treatment strategies until more is known about molecular pathways and driver mutations in glioblastoma.

### Histological diagnosis and classification of malignant glioma subtypes

Histopathology remains the gold standard for the diagnosis and classification of gliomas and currently determines adjuvant therapy. Gliomas include astrocytomas (with cells that resemble astrocytes), oligodendrogliomas (predominantly of cells that resemble oligodendrocytes), and mixed oligoastrocytomas. The current 2007 World Health Organization (WHO) classification of astrocytoma (including glioblastoma) depends on cellular morphology to determine tumor grade, focusing on the presence or absence of nuclear atypia, mitotic activity, microvascular proliferation, and necrosis (1). This histopathological classification system is fraught with error, and inter-observer subjectivity is common (2). Regional heterogeneity is common, as reflected by the original name for WHO Grade IV astrocytoma, glioblastoma multiforme (GBM) (3). Pathological diagnosis is based on the area of the highest grade visible in multiple sections, even though less aggressive areas may also be present in the specimen. Tumors with the same histopathological classification also exhibit widely variable clinical presentation, magnetic resonance imaging (MRI) features, response to therapy and outcome. Primary glioblastoma most commonly presents *de novo* in elderly patients, typically with a short clinical history. In contrast, secondary glioblastoma progresses from lower grade astrocytic lesions, occur in younger patients, are more likely to be located in the frontal lobes, and have a significantly improved outcome (4). Molecular analyses show they arise from different genomic alterations, which may influence response to therapy (5).

## Molecular Classification of Glioblastoma Subtypes

**Figure 1:**
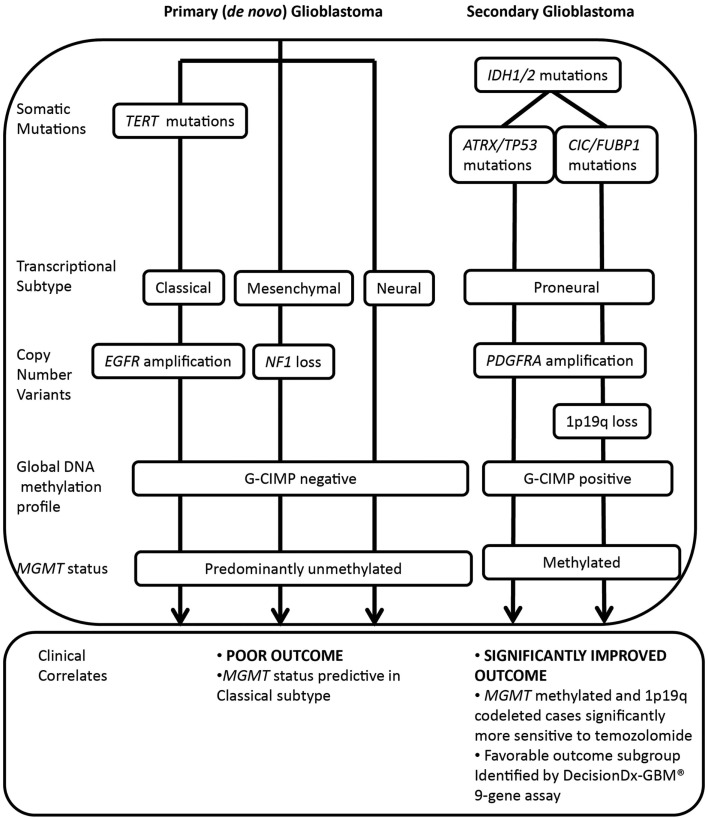
**Molecular classification of major glioblastoma subtypes and correlation with treatment response and outcome**. *IDH1/2* mutations are major prognostic biomarkers, stratifying primary and secondary pathways of gliomagenesis. Primary glioblastoma features a high frequency of *TERT* mutations, whereas *IDH1/2* mutated glioma (including secondary glioblastoma and low grade glioma) may be further subdivided on the basis of co-mutations in either *ATRX* and *TP53* or *CIC* and *FUBP1*, occurring at high frequency in astrocytic or oligodendroglial tumor subtypes. Co-deletion of 1p and 19q, a marker of enhanced chemosensitivity, also clusters with mutations in *CIC* and *FUBP1*. Primary glioblastomas display classical, mesenchymal, and neural phenotypes, whereas secondary glioblastomas tend to display a proneural phenotype that shifts toward a mesenchymal phenotype with recurrence. The significantly improved outcome of the proneural subset is due to the G-CIMP phenotype, established by *IDH1/2*-mediated metabolic reprogramming of the epigenome. Primary glioblastomas and a subset of proneural tumors are glioma-CpG island hypermethylator phenotype (G-CIMP) negative, and a large proportion are *MGMT* unmethylated. Both classical and mesenchymal transcriptional subtypes benefit from concurrent chemoradiotherapy, however, *MGMT* status is only predictive of treatment response in the classical subset.

Recent large-scale genomic analyses have identified extensive inter-patient heterogeneity, further refining histopathological classification of this disease. The Cancer Genome Atlas (TCGA) project and others (5–7) have shown that glioblastoma can be subclassified into at least four molecular subtypes, featuring distinct genetic, epigenetic, and transcriptional alterations (5, 8). Tumor variants can be classified on the basis of somatic mutations in *isocitrate dehydrogenase (IDH) 1/2* and *TP53;* transcriptional signature (classical, mesenchymal, neural, or proneural); copy number variation, including co-deletion of chromosomes 1p and 19q; and amplification or mutation of the *epidermal growth factor receptor (EGFR)* and increased DNA hypermethylation of promoter-associated CpG islands (Figure 1) (5–10).

In particular, the TCGA subtypes have been increasingly applied for their prognostic ability and ease of testing for a single typical molecular alteration associated with that subtype, namely *IDH1/2* mutation (proneural), *EGFR* amplification (classical), or *Neurofibromin 1(NF1)* loss (mesenchymal) (5). Also associated with the proneural subtype are 1p and 19q co-deletion, *TP53* mutations, and *platelet-derived growth factor receptor, alpha polypeptide (PDGFRA)* amplification. These and other molecular aberrations associated with the transcriptional subtypes are shown in Figure 1.

The identified molecular heterogeneity may underlie differences in patient sensitivity to therapy and prognosis. In current clinical practice, standard histopathology is complemented by molecular testing generally performed on single tumor biopsies. Key biomarkers commonly assessed include *IDH1*/2 mutations, *O^6^-methylguanine DNA methyltransferase* (*MGMT*) promoter methylation, co-deletion of 1p and 19q, and *EGFR* amplification/truncation.

### Molecular biomarkers in glioblastoma

#### *IDH1*/*2* mutation status

Somatic mutations in *IDH1/2* are routinely screened by Sanger sequencing and/or immunohistochemistry. These heterozygous point mutations occur commonly at arginine residues, at codon 132 of *IDH1* and at codon 172 of *IDH2*, and are definitive markers of secondary glioblastoma and a significantly improved prognosis (9). *IDH1* mutations occur at high frequency in WHO grade II and III astrocytoma (>80% of cases), precursor lesions of secondary glioblastoma, whereas *IDH2* mutations occur largely in oligodendroglial tumors, with much lower frequency. Both alterations are rare in *de novo* glioblastoma (occurring in <5% of cases). *IDH-*mutant glioblastoma occurs predominantly in the frontal lobe, whereas the anatomical distribution of *IDH*-wildtype glioblastoma is more heterogeneous (11). More recently, loss of *alpha-thalassemia/mental retardation syndrome X-linked (ATRX)* has been shown to further refine *IDH*-mutant astrocytic tumors with *IDH/ATRX* mutant-tumors carrying a more favorable prognosis (10).

In addition to its role as a biomarker, *IDH1* mutation may provide the basis for a targeted immunotherapy. A peptide containing mutant (R132H) IDH1 has been shown to be immunogenic suggesting the potential for a mutation-specific vaccine for *IDH1*-mutant gliomas (12).

In both low- and high-grade gliomas with *IDH* mutations, combined loss of the short arm of chromosome 1 (1p) and the long arm of chromosome 19 (19q; 1p/19q co-deletion) is prognostic of a more favorable outcome than equivalent tumors without this co-deletion (13–15). It is most commonly found in oligodendrogliomas or tumors with oligodendroglial features. The 1p/19q co-deletion and *ATRX* mutations are generally mutually exclusive, *ATRX* mutations being markers for astrocytic lineage tumors (16).

Mutations in *IDH1/2* are also strongly associated with a distinct epigenetic signature. Mutant *IDH1* and *IDH2* alter glioma metabolism, favoring the reduction of α-ketoglutarate to 2-hydroxyglutarate (2-HG) (17), which in turn inhibits DNA and histone demethylases and establishes a glioma-CpG island hypermethylator phenotype (G-CIMP) (5), featuring hypermethylation at a large number of loci. The combination of *IDH* mutation, 1p/19q co-deletion and G-CIMP is prognostic of a favorable outcome for anaplastic oligodendroglial tumors and predictive of chemotherapy response (14, 15). *IDH*-mutant human gliomas also have reduced levels of hypoxia-inducible factor (HIF) 1α and the glycolytic enzyme lactate dehydrogenase A (LDHA) (18). This deficit in glycolytic ability may also contribute to the slower growth of *IDH*-mutant tumors and their improved prognosis.

Most *IDH* mutated glioblastomas feature a proneural transcriptional profile, including a high frequency of *TP53* mutations and amplification of *PDGFRA* (5). In contrast *IDH-*wildtype glioblastoma tends to be subclassified into mesenchymal, classical, and neural transcriptional subtypes. Upon recurrence, proneural tumors shift toward a more aggressive mesenchymal phenotype, enriched for expression of mesenchymal markers including *chitinase 3-like 1* (*CHI3L1* or *YKL40*), *CD44* and *signal transducer and activator of transcription 3* (*STAT3*) (6).

Colman et al. developed a robust 9-gene expression assay (now a proprietary test called DecisionDx-GBM^®^) with the ability to discriminate between glioblastoma patients with a more favorable outcome, associated with high-level expression of proneural genes, and those with a poor outcome, with enriched expression of mesenchymal and angiogenesis genes (19). Prognostic subgroups are identified by profiling tumor RNA extracted from formalin-fixed paraffin embedded (FFPE) tissue blocks and gene expression levels converted to a metagene score, using a proprietary algorithm. Multivariate analyses show that this 9-gene prognostic score is a stronger predictor of overall outcome and progression-free survival than other clinically significant variables, including age, Karnofsky performance score (KPS), and *MGMT*. This 9-gene expression assay independently predicts response to standard first line therapy and has been used to prognostically stratify patients and as a secondary endpoint in two clinical trials (20). However, its lack of validation in larger independent patient cohorts means that it has not been integrated into routine clinical testing.

#### *MGMT* promoter methylation

The current standard treatment for newly diagnosed glioblastoma involves surgery and radiation, in conjunction with the alkylating agent temozolomide (Stupp regimen), achieving a median survival of 14.6 months (21). Response to temozolomide is mediated by *MGMT*, encoding a DNA repair enzyme involved in the repair of cytotoxic adducts produced by this alkylating agent. Hypermethylation or epigenetic silencing of the *MGMT* disables DNA repair capacity, rendering cells more sensitive to treatment (7). *MGMT* promoter methylation is a common feature of *IDH1/2* mutant/G-CIMP positive glioma, however, is less prevalent in G-CIMP negative tumors, such as primary glioblastoma, where *MGMT* methylation occurs in approximately 40% of cases (22–24).

Several clinical trials, including the landmark studies by Stupp and Hegi have shown that a methylated *MGMT* promoter is an independent predictor of response to therapy and outcome (21, 25–28). Patients with a methylated *MGMT* promoter exhibited a 6.4-month survival benefit following concurrent treatment with temozolomide and radiotherapy, compared to cases receiving radiotherapy alone (25), whereas the benefit for unmethylated cases was less than 1 month (25). The NOA-08 and Nordic clinical trials in elderly patients presenting with primary glioblastoma have also shown that a methylated *MGMT* promoter is predictive and prognostic in response to therapy (29, 30). The Nordic study found that patients more than 60 years of age with methylated *MGMT* gained an overall survival benefit following temozolomide therapy, whereas methylation status did not correlate with overall survival following radiotherapy (30). In the NOA-08 trial, patients more than 65 years of age with methylated *MGMT* and a favorable Karnofsky performance status similarly showed a greater overall survival benefit following dose dense temozolomide therapy than patients with unmethylated *MGMT* (29).

However, the use of *MGMT* methylation as a predictive and prognostic biomarker in the routine clinical environment is not straightforward. Some patients with methylated *MGMT* do poorly, and occasionally patients with unmethylated *MGMT* have prolonged survival. This is likely due to additional molecular changes. For example, it has recently been reported that *MGMT* methylation status was only predictive in glioblastoma cases with a classical transcriptional gene signature (7). There is also evidence that defects in the mismatch repair (MMR) pathway can confer increased resistance to temozolomide independent of *MGMT* methylation status (31) with mutations in *mutS homolog 6* (*MSH6*) (32) and aberrant expression of MMR proteins MSH6, mutS homolog 2 (MSH2), PMS2 postmeiotic segregation increased 2 (PMS2), and mutL homolog 1 (MLH1) reported in some glioblastoma specimens (33, 34). The lack of correlation between *MGMT* status and treatment response in some cases may be related to technical aspects of current analytical assays. Differences in the efficiency of the initial bisulfite conversion step and also the region of the *MGMT* promoter selected for methylation analysis may contribute to variability in *MGMT* results. Everhard et al. performed a comprehensive study of CpG islands with the *MGMT* promoter, correlating methylation status with *MGMT* mRNA levels in 54 glioma specimens (35). This analysis identified 6 out of the 52 CpG sites as having the strongest correlation with *MGMT* expression (*p* < 0.0001), indicating that methylation at some sites may be more informative than others. MGMT expression may be induced by glucocorticoids (36), cAMP and protein kinase C activators, and radiation to a moderate extent (37, 38). Detection of MGMT expression by immunohistochemistry has proved unreliable in predicting response (25, 39, 40).

#### *EGFR* amplification/truncation

*EGFR* is an attractive therapeutic target in glioblastoma, with gene amplification noted in 40–60% of patients (1, 41, 42). A constitutively active mutation of *EGFR* (*EGFRvIII*) is found in 20–30% of glioblastomas and typically occurs in the presence of over-expression (amplification) of the wild type transcript. *EGFRv111* is an in-frame genomic deletion of exons 2–7 resulting in a truncated protein with constitutive tyrosine kinase activity, pro-oncogenic effects, and increased chemotherapeutic and radiotherapy resistance. It is an independent marker of poor prognosis in glioblastoma (42–44).

*EGFR* alterations increase cell signaling through multiple pathways, including the phosphatidylinositol-3-kinase (PI3K)/Akt/mTOR pathway, and ultimately accelerate tumor growth and progression. However, attempts to block EGFR signaling in the clinic using small molecule inhibitors, such as erlotinib and gefitinib, or monoclonal antibodies, such as cetuximab have been largely unsuccessful, even after molecular preselection of patients (45). An alternate strategy targeting EGFRv111 with a vaccine (Rindopepimut) (46) is currently in Phase 3 clinical trials for patients with newly diagnosed (ACT IV) and with relapsed (ReACT) *EGFRv111*-positive glioblastoma following promising phase II trials (47, 48).

Focal homozygous deletion of *cyclin-dependent kinase inhibitor 2A (CDKN2A)* is a frequent finding in *EGFR* amplified tumors and these, together with the *EGFRvIII*, are features of the classical transcriptional subtype of glioblastoma (5). More recently, the presence of activating *telomerase reverse transcriptase* (*TERT)* promoter mutations have been reported in over 80% of primary glioblastomas. In this cohort, *EGFR* amplification occurred only in *TERT*-mutated tumors (49).

## Limitations of Single-Biopsy Based Diagnosis in Glioblastoma

Despite the major advances in the molecular profiling of glioblastoma, improvements to patient outcome overall have been modest. Patient stratification and treatment have generally been performed on the basis molecular biomarkers present in a single tumor specimen. However, this approach may be too simplistic, as recent studies have unraveled yet further layers of complexity, with striking molecular heterogeneity also present within individual tumor specimens. Sottoriva et al. (50) employed a novel fluorescence-guided multiple sampling approach to collect spatially distinct tumor fragments from 11 glioblastomas, demonstrating the presence of multiple transcriptional tumor subtypes within the same tumor mass (50). Spatial heterogeneity in glioblastoma not only confounds histopathological classification but can also make treatment decisions based on samples obtained from limited areas difficult, especially as we enter the realm of personalized medicine and targeted therapies (2, 51, 52). There is also evidence of temporal heterogeneity, with selective expansion or regression of particular tumor cell subpopulations resulting from various treatments (53).

The sampling of glioma presents a challenge unique to brain tumors (54–56). Extracranial tumors can usually be resected en-bloc to preserve the geographical map from which spatially distinct tumor areas can be characterized and tumor margins and draining lymph nodes examined to assess extent of disease. Some gliomas are located in eloquent areas limiting sampling to a single biopsy. Larger gliomas are generally resected in a piecemeal fashion in order to minimize damage to surrounding brain and preserve function and quality of life of the individual. Most surgery is guided by gadolinium enhanced MRI scans. High-grade tumors are usually resected up to and including the macroscopically abnormal margin or to the area of contrast enhancement on T1 weighted MRI. It is well known, however, that aggressive infiltrating malignant cells lie well beyond this definable margin (54–56). Newer techniques using fluorescent dyes such as 5-aminolevulinic acid (ALA) potentially allow surgeons to identify areas of residual high-grade tumor at surgical margins making it possible to remove more tumor, prolonging patient progression-free survival (57).Using newer guidance technology may be able to separate these tumors spatially for analysis and potentially provide better guides to therapy. However, it is not clear from which location and how many biopsies constitute a *bona fide* representative sample. The co-existence of genetically divergent tumor cell clones may lead to a misinterpretation of the overall genetic tumor landscape if only a single sample is utilized.

## Tumor Heterogeneity

### Theories of tumor heterogeneity

**Figure 2:**
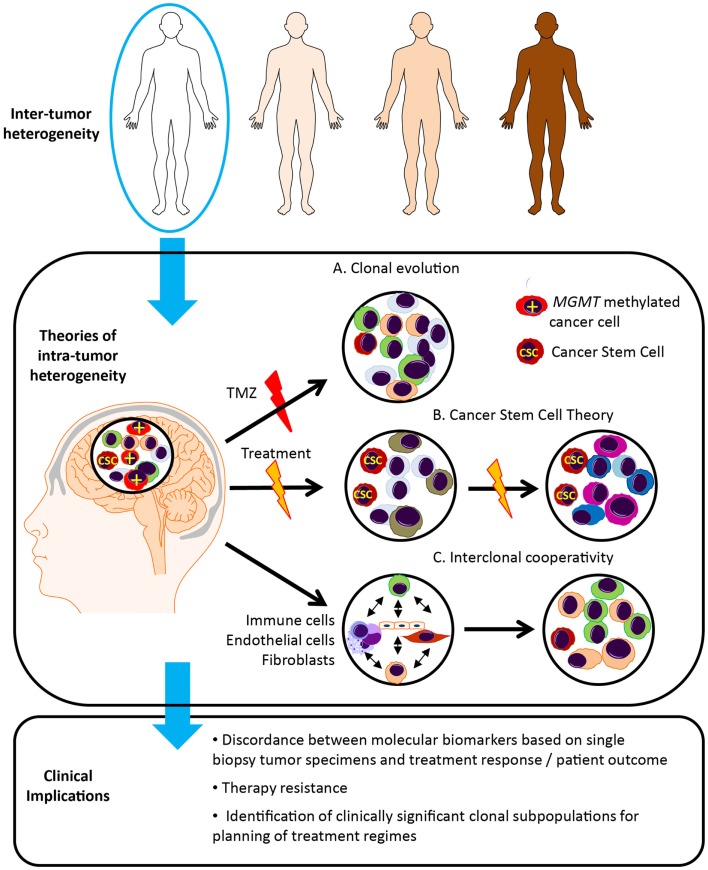
**Glioblastoma tumor heterogeneity and implications for patient management**. Tumor evolution and tumor heterogeneity may be promoted by clonal evolution, cancer stem cells and interclonal cooperativity. (A) According to the theory of clonal evolution, somatic alterations affecting the initial cell of origin give rise to multiple cancer clones, with different sensitivity to therapy and ability to survive and proliferate. These tumor cell clones are genetically unstable, undergoing successive waves of genetic alterations, and clones with the most aggressive phenotype are favored. For example, only the *MGMT* methylated cells sensitive to temozolomide (TMZ) disappear following TMZ treatment. (B) In contrast, according to the cancer stem cell theory, only a single subset of cells, known as cancer stem cells (CSC), possess the ability to self-renew, continuously proliferate and give rise to clones of variable genetic profiles, and are inherently resistant to therapy. (C) The theory of interclonal cooperativity suggests that tumor evolution and heterogeneity is promoted by interactions between tumor cell clones and their microenvironment, with immune/stromal factors influencing malignant progression. Significant clonal diversity within tumor specimens may explain the failure of molecularly targeted therapies in glioblastoma patients.

There are a number of theories regarding tumor evolution and the generation of heterogeneous tumor cell subpopulations (Figure 2). In the Darwinian theory of clonal evolution and natural selection, successive waves of tumor cells acquire genetic changes, some of which convey a proliferative advantage (58). Selection and proliferation of therapy resistant clones lead to tumor progression and resistance to therapy (59). Such branched tumor evolution underscores the importance of targeting ubiquitous alterations in the trunk of the phylogenetic tree (60). In this theory, “driver mutations” allow the progression of a cancer, whereas “passenger mutations” are neutral or only slightly deleterious (61). More recently, with the use of high-resolution “next-generation” sequencing platforms, the definition of driver events has been further refined to encompass “Mut-driver genes” and “Epi-driver genes.” (62). Mut-driver genes tend to display a non-random mutation pattern and are recurrently mutated at the same amino acid position (for example, *IDH*). Epi-driver genes may be aberrantly expressed as a result of gene amplification/loss or epigenetic alterations. These early somatic events drive tumor growth and can be considered as “trunk events,” with branched separation of tumor subclones giving rise to tumor heterogeneity (63). This theory has been studied in colon cancer where the evolution of genomic changes from benign polyps to invasive carcinoma can be tracked from colonic biopsies. Study of secondary glioblastoma, which usually develops from *IDH* mutated low-grade lesions to invasive glioblastoma, may be possible, however primary glioblastoma appears to develop rapidly, with some patients having had a normal MRI just months before presentation. This group poses a particular challenge.

An alternative theory is the cancer stem cell theory, which postulates that only a subset of tumor cells possess the ability to self-renew, continuously proliferate and give rise to clones containing variable genetic profiles, and the heterogeneity within a tumor arises from proliferation of these genetically variable cancer stem cells (64, 65). Genetically distinct cancer clones may originate in specific anatomical locations, differing in neurochemistry, energy metabolism, and surrounding tissue architecture (66). Gliomas are thought to originate in the subventricular zone, which contains the highest density of astrocyte-like stem cells, and may be more susceptible to malignant transformation (56). Distinct tumor clones may harbor genetic alterations that promote invasion along blood vessels and white matter tracts and tumor expansion in particular cerebral lobes. The anatomical distribution of glioblastoma in the brain is heterogeneous, occurring with the highest frequency in the frontal lobe, followed by the temporal, parietal and occipital lobes (66).

More recently a third theory, “interclonal cooperativity” has emerged in which genetic subpopulations of tumor cell clones and immune/stromal factors co-operate to create a favorable microenvironment (67–69). Feedback from the microenvironment drives disease progression and a malignant phenotype. The complex tumor stroma, consisting of reactive astrocytes, microglial cells and immune infiltrate, aberrant microvascular proliferation and hypoxia, as well as cell populations of varying developmental stages, may also enhance the proliferation of specific cancer subclones. Specifically, the hypoxic perivascular niche has been shown to be critical for the self-renewal of glioblastoma cancer stem-like cells (70, 71), and may be important in driver of divergent tumor evolution and treatment resistance.

Unlike other tumors, gliomas rarely metastasize outside the brain and despite intense local radiotherapy, most commonly recur at the site of origin. Whether these recurrent tumors arise from incompletely resected primary tumor or distinct cancer clones that have evolved in response to treatment or other factors, such microenvironmental cues, is unknown. However, a recent whole exome sequencing study of low grade glioma supports the concept that recurrent disease originates from tumor cells present at tumor initiation that subsequently drive evolution of the tumor (72), with just under half of recurrent specimens sharing 50% of mutations with the primary tumor sample.

### Evidence of molecular intra-tumoral heterogeneity in glioblastoma

As described above, microscopic heterogeneity is a hallmark of glioblastoma. The evolution of more detailed molecular investigation and their potential implication in treatment decision making has resulted in the further verification of tumor heterogeneity at the molecular level. Tumor development and evolution has also been extensively investigated and Sottoriva et al. have demonstrated tumor heterogeneity in tumor evolution (50). Further to this, Meyer et al. by establishing *in vitro* functionally distinct clonal populations from glioblastoma specimens, have demonstrated the potential differential response to treatment of these various clones (73).

Both intra-tumoral and temporal heterogeneity in *MGMT* status have been reported in some (74) but not all (75) such investigations in glioma. Several authors have also demonstrated intra-tumoral heterogeneity of receptor tyrosine kinases (RTKs) in gliomas (76–78). Snuderl et al. (76) identified intermingled populations of tumor cells containing varying amplification of up to three different RTK genes (*EGFR*, *MET*, and *PDGFRA*), all of which were derived from a common precursor. Likewise, Szerlip et al. (77) found that multiple RTKs were likely to be maintaining distinct cell subpopulations. Little et al. (78) also observed a high degree of variability in gene copy number of *EGFR* and *PDGFRA* in individual cells across entire glioblastoma specimens.

More recently, Patel et al. performed single cell RNA sequencing on 430 cells from five primary glioblastomas, documenting intra-tumoral variation in the expression of a range of transcriptional programs, including oncogenic signaling, proliferation, immune response, and hypoxia (79).

### Clinical implications of molecular heterogeneity in glioblastoma

Intra-tumoral heterogeneity has important implications for the development of prognostic and predictive biomarkers and their ability to guide personalized treatment regimens. Transcriptional profiling of glioblastoma into molecular subtypes is based on the average expression of genes across a sample irrespective of intra-tumoral heterogeneity (80) and may limit the usefulness of prognostic panels, such as DecisionDx-GBM^®^. Similarly, intra-tumoral molecular heterogeneity may underlie discordance between predictive markers, such as *MGMT* and treatment response.

Intra-tumoral heterogeneity may also contribute to the failure of targeted therapies in these patients. Glioblastomas possess genetic alterations that target three core signaling pathways, including the RTK/RAS/PI3K axis, p53/MDM2/MDM4 axis, and RB/CDK4/INK4A axis (7). However, targeting of these pathways with monotherapies (such as the first generation EGFR kinase inhibitors gefitinib and erlotinib) has failed to achieve any significant clinical benefit in the majority of patients (81, 82). Diversity of tumor subclones within the same individual, together with poor drug penetration and activation of other compensatory pathways in response to RTK inhibition (83, 84) is likely to account for this negative result. Molecular preselection of patients for clinical trials is challenging and tumor-sampling methods need to consider the regional diversity of tumor subclones. Technology is available to sequence genetic material from FFPE tumor blocks, however, information on the spatial orientation of specimens may not be available (80). Treatment of patients with more than one agent, targeting major clones may be possible but limited by drug toxicity and the ability of agents to penetrate the blood brain barrier (80).

The development of new treatment strategies for recurrent disease must consider temporal diversity of tumor clones, with molecular biomarkers assessed in recurrent tumor biopsies where possible, since they may differ substantially from initial diagnosis. Tumors harbor a complex hierarchy of clones, the proportions or dominance of which may change following therapy. Molecular biomarkers that are prognostic/predictive at initial diagnosis may not be informative at recurrence. For example, the TCGA transcriptional subtypes were determined using a cohort of primary biopsies and may not be robust classifiers of recurrent disease.

Monitoring of clonal dynamics during treatment and targeting of drug resistance clones may significantly improve patient outcomes. However, this may be difficult to implement in the clinic, with extensive/serial debulking not an option for glioblastoma patients. Serial measurements of circulating tumor DNA for tumor specific markers such as *EGFRv111* (85), and the use of imaging modalities to non-invasively monitor molecular biomarkers may allow clinicians to monitor clonal evolution and treatment response.

Although major advances in sequencing approaches are enabling tumor heterogeneity to be studied at unprecedented resolution, the interpretation of results is still evolving, with the detection and the clinical significance of minor clones still in question. For example, heterogeneity may in some cases lead to treatment resistance. Clinically actionable mutations must be verified by independent analysis using an alternative technology, such as Sanger sequencing (86); however, this technology is limited by its sensitivity and may not detect important low frequency variants. Clinicians need to decide which mutations to prioritize for the purpose of treatment design. Alternatively, a measure of subclonal diversity, such as the Shannon index, which estimates the total number of tumor subclones and the relatively frequency of each clone, may itself may be a useful prognostic biomarker, as has been shown in the case of esophageal adenocarcinoma (87).

## Conclusion

Our increased knowledge of molecular alterations in glioblastoma has improved patient diagnosis and identified many new potential therapeutic targets. Treatments directed at these specific alterations may improve patient outcomes and reduce toxicity. The introduction of molecular testing into routine diagnostic pathology will also enable clinicians to better stratify patients for clinical trials, selecting those that may benefit from targeted therapy.

While molecular profiling allows us to explore in depth, the biological characteristics of a tumor, performing this on a single biopsy specimen may be overly simplistic and inadequately reflect both the genomic landscape and overall behavior of a tumor. It is being increasingly recognized that glioblastoma contain multiple distinct populations of tumor cells with the potential to convey survival advantage and resistance to therapy, and these may be selected and enriched through successive cycles of treatment. However, many trials are conducted in relapsed patients whose tumors may have been altered by previous radiotherapy and alkylating agents which are known to cause additional mutations, with molecular preselection based on their original pathology specimen. Intra-tumoral heterogeneity, together with the concomitant activation of multiple oncogenic signaling pathways in glioblastoma, suggests that a single agent will be ineffective in treating this disease. Importantly, next generation sequencing technology may enable the identification of molecular alterations occurring early in gliomagenesis, the so-called “driver events,” which may represent better targets for the design of therapeutic regimes.

Glioblastoma poses some unique challenges to clinicians – tumor cells diffusely invade surrounding normal brain parenchyma, making complete surgical resection impossible, and the efficacy of therapeutic agents are often limited by the blood brain barrier. Resampling of tumors following treatment is also limited. The development of non-invasive imaging techniques, such as those associated with PET scanning, and identification of circulating tumor cells harboring molecular biomarkers may enable clinicians to non-invasively monitor clonal dynamics during treatment and the development of disease recurrence.

The link between glioblastoma molecular subtype and response to treatment is still incompletely understood. While the answer may be gleaned from the molecular analysis of multiple biopsies at each initial and subsequent resection, the use of such information is still yet to prove beneficial in conveying any meaningful survival advantage to those suffering from glioblastoma.

## Conflict of Interest Statement

The authors declare that the research was conducted in the absence of any commercial or financial relationships that could be construed as a potential conflict of interest.
